# Molecular Cloning of a Novel Glucuronokinase/Putative Pyrophosphorylase from Zebrafish Acting in an UDP-Glucuronic Acid Salvage Pathway

**DOI:** 10.1371/journal.pone.0089690

**Published:** 2014-02-28

**Authors:** Roman Gangl, Robert Behmüller, Raimund Tenhaken

**Affiliations:** 1 Department of Cell Biology, Division Plant Physiology, University of Salzburg, Salzburg, Austria; 2 Department of Molecular Biology, Division of Chemistry and Bioanalytics, University of Salzburg, Salzburg, Austria; Institute of Enzymology of the Hungarian Academy of Science, Hungary

## Abstract

In animals, the main precursor for glycosaminoglycan and furthermore proteoglycan biosynthesis, like hyaluronic acid, is UDP-glucuronic acid, which is synthesized via the nucleotide sugar oxidation pathway. Mutations in this pathway cause severe developmental defects (deficiency in the initiation of heart valve formation). In plants, UDP-glucuronic acid is synthesized via two independent pathways. Beside the nucleotide sugar oxidation pathway, a second minor route to UDP-glucuronic acid exist termed the myo-inositol oxygenation pathway. Within this myo-inositol is ring cleaved into glucuronic acid, which is subsequently converted to UDP-glucuronic acid by glucuronokinase and UDP-sugar pyrophosphorylase. Here we report on a similar, but bifunctional enzyme from zebrafish (*Danio rerio*) which has glucuronokinase/putative pyrophosphorylase activity. The enzyme can convert glucuronic acid into UDP-glucuronic acid, required for completion of the alternative pathway to UDP-glucuronic acid via myo-inositol and thus establishes a so far unknown second route to UDP-glucuronic acid in animals. Glucuronokinase from zebrafish is a member of the GHMP-kinase superfamily having unique substrate specificity for glucuronic acid with a K_m_ of 31±8 µM and accepting ATP as the only phosphate donor (K_m_: 59±9 µM). UDP-glucuronic acid pyrophosphorylase from zebrafish has homology to bacterial nucleotidyltransferases and requires UTP as nucleosid diphosphate donor. Genes for bifunctional glucuronokinase and putative UDP-glucuronic acid pyrophosphorylase are conserved among some groups of lower animals, including fishes, frogs, tunicates, and polychaeta, but are absent from mammals. The existence of a second pathway for UDP-glucuronic acid biosynthesis in zebrafish likely explains some previous contradictory finding in *jekyll/ugdh* zebrafish developmental mutants, which showed residual glycosaminoglycans and proteoglycans in knockout mutants of UDP-glucose dehydrogenase.

## Introduction

The skeleton of vertebrates provides structural support for muscle attachments and a protection of internal organs [1]. These functions rely on the coordinated secretion of extracellular matrix (ECM) by skeletal precursor cells during embryonic development [1]. ECM includes collagen, which anchors and reinforces the ECM, elastin, which provides flexibility [2], and proteoglycans, which impact cell division, cell adhesion, and migration [3], [4], [5], [6]. Glycosaminoglycan (GAG) and furthermore proteoglycan (PG) biosynthesis requires UDP-glucuronic acid (UDP-GlcA) as a common carbohydrate precursor found in hyaluronic acid, chondroitin, dermatan and heparin. The enzyme UDP-glucose dehydrogenase (UGDH) oxidises UDP-glucose to UDP-GlcA and provides the cell with this important nucleotide sugar.

In zebrafish, skeletal development can be disrupted by mutations at several steps in the pathway of GAG and PG biosynthesis [1]. *jekyll/ugdh* zebrafish mutants with diminished UGDH activity show defective craniofacial and coronary development. j*ekyll/ugdh* zebrafish mutants are deficient in the initiation of heart valve formation [7], [8]. In addition, mice deficient for hyaluronan synthase 2 (EC 2.4.1.212, HAS2) [9] also exhibit *jekyll/ugdh mutant*-like valve defects [10], suggesting that UGDH may also function in valve formation through its requirement for hyaluronic acid (HA) synthesis. *dfna5* zebrafish morphants lead to disorganization of the developing semicircular canals and reduction of pharyngeal cartilage [11]. The phenotype seen in *dfna5* zebrafish morphants closely resembles the *jekyll/ugdh* zebrafish mutant phenotype [11], [12]. Based on the similar phenotypes, it was hypothesized that DFNA5 may be involved in the UGDH pathway [11]. In *dfna5* zebrafish morphants, expression of UGDH is absent in the developing ear and pharyngeal arches, and HA levels are strongly reduced in the outgrowing protrusions of the developing semicircular canals [11]. *jekyll/ugdh* zebrafish mutants are characterized by very weak but still visible cartilage staining with Alcian blue [1]. In contrast, a mutant in UDP-xylose synthase (*uxs1*) in zebrafish totally lacks cartilage staining with Alcian blue at PG rich ECM in cartilages of the neurocranium, pharyngeal arches and pectoral girdle [1]. As UDP-xylose synthase uses UDP-GlcA as a substrate to form UDP-xylose after decarboxylation, one would expect very similar staining patterns in both mutants, because the enzyme UGDH is upstream of the *uxs1* mutant. One explanation for this incongruity could be that another protein/pathway from zebrafish partially compensates for the lack of UGDH [8].

**Figure 1:**
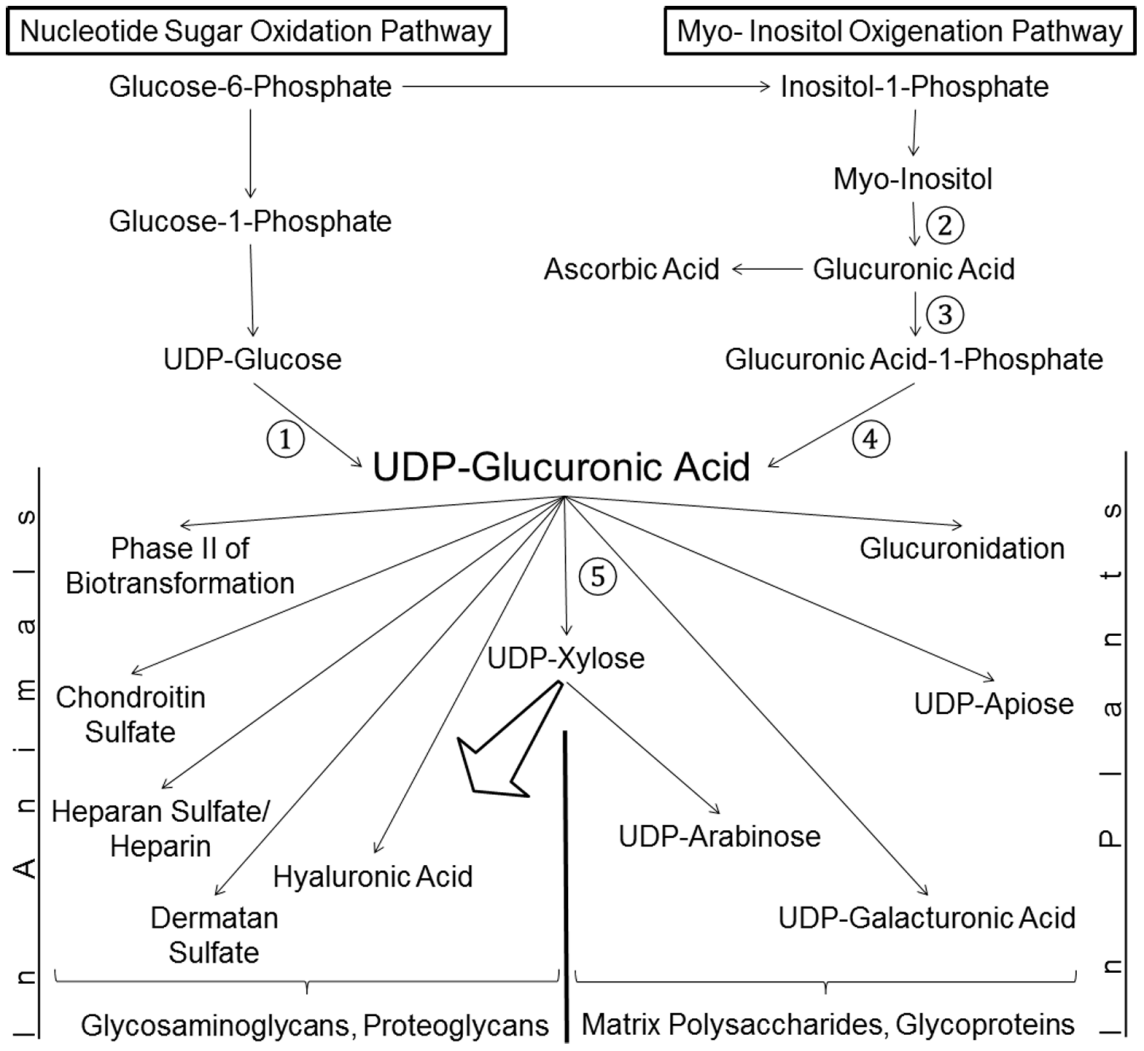
Biosynthesis of UDP-GlcA in Lower Animals. Two pathways for the biosynthesis of UDP-GlcA are present in plants and lower animals. The nucleotide sugar oxidation pathway with the enzyme UDP-glucose dehydrogenase (1) is predominant in both plant and animal kingdoms. Glucuronokinase (3) and UDP-glucuronic acid pyrophosphorylase (4) are part of the myo-Inositol oxygenation pathway starting with myo-inositol oxygenase (2). UDP-xylose synthase (5) converts UDP-GlcA into UDP-xylose for GAG and furthermore PG biosynthesis. Note that GlcA is also the precursor for ascorbic acid in animals. Thus the salvage pathway to UDP-GlcA and ascorbic acid biosynthesis compete for the same substrate GlcA.

In animals and plants UDP-GlcA is predominantly or exclusively synthesized by UGDH. The biochemical pathway is shown in Fig. 1. Early studies by [13] pointed out the existence of a second pathway in plants beside UGDH which is called the myo-inositol oxygenation pathway. Within this, myo-inositol is converted into glucuronic acid (GlcA) by an O_2_-dependent ring cleavage. GlcA is converted to UDP-GlcA via an intermediate step, the synthesis of GlcA-1-phosphate by glucuronokinase (EC 2.7.1.43) [14] followed by a pyrophosphorylase reaction (Fig. 1). This pathway is believed to be present in many if not all plants but was never reported to be functional in animals. Out of the three enzymatic steps required from myo-inositol to UDP-GlcA only the enzyme myo inositol oxygenase is widely present in animals [15]. The purification and cloning of glucuronokinase from Arabidopsis plants [16] gave a hint that a similar enzyme might be present in some lower animal groups like fishes and amphibians.

In plants up to now sugar-1-kinases were identified for monomers of arabinose [17], galactose [18], fucose [19], galacturonic acid [20] and glucuronic acid [16]. The corresponding sugar-1-phosphates - except for fucose – are converted subsequently to UDP-sugars by a common UDP-sugar pyrophosphorylase [21]. All known sugar-1-kinases belong to the group of GHMP-kinases (name giving galacto-, homoserine-, mevalonate-, and phosphomevalonate-kinases), which are characterized by special ATP-binding site and their ability to phosphorylate diverse low molecular weight compounds.

The plant UDP-sugar pyrophosphorylase [21] has a broad substrate specificity and a similar enzyme is also present in trypanosomes [22] and archaea [23] but seems to be absent from all animals. Instead, the glucuronokinase from zebrafish contains a 30 kDa N-terminal domain with homology to bacterial nucleotidyltransferases.

Nucleotidyltransferases vary in length: in eukaryotes, UDP-glucose pyrophosphorylase ranges in size across species from 470 to 510 amino acids, whereas UDP-glucose pyrophosphorylase in prokaryotes are much shorter at about 300 amino acids [22], [23], [24]. Interestingly the zebrafish enzyme contains a short pyrophosphorylase domain that shares homology to bacterial enzymes.

Here we report on the characterization of a *Danio rerio* glucuronokinase with putative uridyl pyrophosphorylase (DrGKUP) activity. In some experiments the function of single domains e.g. the glucuronokinase, was studied. Consequently, this recombinant protein, consisting of glucuronokinase domain was named DrGK. If only the putative uridyl pyrophosphorylase domain was present we named the protein DrUP.

## Materials and Methods

### cDNA Cloning

EST-cDNA clone (IMAGE: 9005473, EH998169.1) from *Danio rerio* was purchased from Source BioScience, Nottingham, UK. We received first strand cDNA of *Xenopus tropicalis* tadpoles from Prof. Dr. Christof Niehrs (University of Heidelberg and DKFZ Heidelberg, Germany). For cloning the DrGKUP, DrGK and DrUP gene from zebrafish and the *Xenopus tropicalis* (XtGKUP) primers with appropriate restriction sites were designed based on the cDNA sequence of zebrafish or claw frog (primer sequences are given in Table S1). PCR was performed with Phusion High-Fidelity DNA polymerase (New England Biolabs) using primer pairs and single-stranded cDNA as template under the following conditions: 30 s initial denaturation at 98°C, 10 s denaturing at 98°C, 30 s annealing at T_A_ (Tab. S1), either 45 s or 90 s elongation at 72°C for 35 cycles and 5 min final extension at 72°C. The amplified PCR-products were gel purified, cut with appropriate enzymes (Fast Digest; Thermo) and ligated into the bacterial (pET32), yeast (pToy; [25]) or plant (pGreen0229 with CaMV35 –Strep Tag cassette) expression vectors.

### Transient Transformation of tobacco (*Nicotiana benthamiana*) leaves

For expression of recombinant protein, pGreen0229 vectors containing either DrGKUP, DrGK, DrUP or XtGKUP was transfected together with the helper plasmid pSoup into electro competent *Agrobacterium tumefaciens* (GV3101 (pMP90)) [26]. To prevent gene silencing, an Agrobacterium expressing the viral p19 repressor [27] was coinfiltrated in tobacco leaves.

Bacteria were grown in YEB medium supplemented with 50 µg·ml^−1^ kanamycin and 25 µg·ml^−1^ gentamycin, and grown in an incubator overnight at 28°C under continuously shaking, about 200 rpm, to the late exponential phase. *A. tumefaciens* culture was harvested by centrifugation and pellets were resuspended in infiltration buffer (10 mM MES-KOH (pH 5.6), 10 mM MgCl_2_, 150 µM acetosyringone). *A. tumefaciens* cultures carrying the gene of interest were adjusted to OD_600_ of 0.5 with infiltration buffer and *A. tumefaciens* culture expressing p19 repressor were diluted to OD_600_ of 0.25. The suspensions were incubated for 2 hours at RT and finally, both cultures were mixed in the ratio of 1 to 1 before infiltration of four weeks tobacco leaves. Infiltrated leaf areas were marked with a pen, to allow precise recognition of the infiltrated leave areas and harvested after 7–10 days at short day conditions (10 h light, 14 h darkness) for recombinant protein purification.

### Purification of Recombinant Protein from tobacco leaves

Infiltrated leaf areas were homogenized in a precooled mortar with 1.5 ml extraction buffer (100 mM HEPES (pH 8.0), 100 mM NaCl, 5 mM EDTA (pH 8.0), 15 mM DTT, 0.5% Triton X-100, 100 µg·ml^−1^ avidin) for each 0.75 g leaf material. The remaining frozen leaf material was stored at −80°C. While thawing on ice the crude extract was vortexed vigorously and incubated for 10 min on ice. Cells were additionally disrupted by sonication with an ultrasonic needle (60% ultrasonic-capacity pulse: 10 sec, three times, 10 sec pause between pulses) and cell debris was pelleted by centrifugation at 9,000× g for 10 min. The supernatant was centrifuged again and the residual supernatant was filtered through two layers of Miracloth (Calbiochem/Merck) to remove any residual solid particles. The crude extract was applied to 40 µl of Strep-Tactin Macro Prep (50% suspension; IBA BioTAGnologies, Göttingen, Germany) equilibrated in 100 µl extraction buffer for 20 min on a rotation wheel at about 15 rpm. The crude protein extract was incubated for 10 min on the rotation wheel, centrifuged at about 2,200 rpm and the supernatant was removed. Strep-Tactin Macro Prep resin was washed 5 times using 500 µl wash buffer (100 mM HEPES (pH 8.0), 100 mM NaCl, 0.5 mM EDTA (pH 8.0), 2 mM DTT, 0.005% Trition X-100) and incubated 10 min on the rotation wheel, centrifuged and the supernatant was removed. Bound recombinant protein was eluted twice by incubating resin in 75 µl elution buffer (100 mM HEPES (pH 8.0), 100 mM NaCl, 0.5 mM EDTA (pH 8.0), 2 mM DTT, 0.005% Trition X-100, 10 mM desthiobiotin) for 5 min on thermo mixer, at about 1,000 rpm. The recombinant protein solution was portioned in 50 µl aliquots in 0.5 ml reaction tubes with 20% glycerol, immediately frozen in liquid nitrogen and stored on −80°C for long term storage.

### Expression and Purification of Recombinant Protein from *E. coli* cells

For expression of recombinant protein from *E. coli*, *DrGKUP* was cloned into a pET32-c vector with an N-terminal His_6_-tag. The expression construct was transformed into either BL21 cells or the Origami™ strain (Novagen) of *E. coli* and cultivated in a 250 ml Erlenmeyer flask containing 50 ml of liquid LB medium supplemented with antibiotics. The culture was shaken with 200 rpm at 37°C for several hours until an OD_600_ of 0.6 was reached. The main culture was cooled on ice to 18°C and the expression of recombinant protein was induced by addition of 1 mM Isopropyl-β-D-thiogalactopyranoside (IPTG) overnight at 18°C with continuous shaking. All following purification steps were carried out at 4°C. The recombinant protein was purified with a Protino-Ni1000 column (Macherey & Nagel, Düren, Germany) according to the manufacturer's protocol. Recombinant protein solution was desalted on a PD-10 column (GE Healthcare) into storage buffer (20 mM Tris/Cl (pH 8.7), 50 mM KCl, 20% (v/v) glycerol) immediately frozen in liquid nitrogen and stored on −80°C for long term storage.

### Western Blot Detection

Different fractions, obtained during strep-tag purification of transiently expressed glucuronokinase (either DrGKUP, DrGK, DrUP or XtGKUP) were separated by SDS-PAGE and transferred on a PVDF-membrane. Blocking of non-specific binding sites was done by incubating the membrane with TBST-1% BSA solution for 1 h and 10 min incubation in TBST with 2 µg·ml^−1^ avidin. After addition of Strep-Tactin alkaline phosphatase conjugate (IBA BioTAGnology) in a dilution of 1∶50000, the membrane was further incubated for 1 h at RT. Unbound conjugate was removed by washing the blot with TBST 4 times for 5 min. Detection of alkaline phosphatase was performed with CDP-Star reagent (New England Biolabs) on a LAS-3000 imaging system (Fuji). Detection of His-tagged proteins was carried out using SuperSignal® West HisProbe™ Kit (Thermo Scientific).

### Protein Determination

Protein concentration was determined on the NanoDrop® ND-1000 Spectrophotometer using the method of Bradford assay with bovine serum albumin as reference protein.

### Expression of Recombinant Protein in *S. cerevisiae* cells

For expression of recombinant protein, a pToy4 vector containing the DrGKUP cDNA was transformed in W303 *YEL060c* (Δprotease) yeast strain. Yeast cells were grown in 250 ml Erlenmeyer flask containing 50 ml of liquid SC drop-out (-ura) medium supplemented with 5 g·l^−1^ glucose and 5 g·l^−1^ glucuronic acid grown in an incubator at 28°C with continuously shaking, about 200 rpm, for several hours until an OD_600_ of 0.6 was reached.

### Chloroform-Methanol Extraction of UDP sugars

Yeast cells, 50 ml, were harvested by centrifugation at 5,000× g for 2 min at RT and the pellet was resuspended in 250 µl of the quenching solution (chloroform∶methanol; 3 to 7 ratio) with glass beads. The samples were vortexed at 4°C for 15 min, incubated at −20°C for 2 hours and vigorously mixed every 30 min. UDP-sugars were extracted twice by adding 400 µl of water and the aqueous layer was collected after centrifugation at 13,000× g for 10 min [28]. The aqueous phase containing the UDP-sugars was vaporized by a concentrator 5301 (Eppendorf) at 30°C using the setting 3, which is recommended for aqueous solutions and finally reconstituted in 1 ml water.

### Solid phase extraction of UDP Sugars

Samples were applied to Supelclean™ ENVI™-Carb SPE Tubes (3 ml, 0.25 g, particle size: 120–400 mesh) (Supelco) equilibrated with 3 ml equilibration-elution buffer (60% acetonitrile in water containing 0.3% formic acid adjusted to pH 9 with ammonia) and flushing with 3 ml of water. After sample application, washing steps were performed, first with 3 ml of water and afterwards with 1 ml wash buffer (60% acetonitrile in water). Sample elution was done by flushing the column with 2 ml equilibration-elution buffer [29]. The aqueous phase containing the UDP-sugars was vaporized by under vacuum in a concentrator 5301 (Eppendorf) at 30°C and reconstituted in 100 µl water.

### Yeast Protein Extraction

Cells were harvested by centrifugation at 5,000× g for 2 min at RT and the pellet was resuspended in 100 µl distilled water. 100 µl of 0.2 M NaOH was added and incubated at RT for 5 min. Cells were centrifuged at 13,000× g for 2 min at RT and the supernatant was removed. The pellet was resuspended in 50 µl SDS-Page sample buffer, boiled at 95°C for 3 min and centrifuged again. 20 µl of the supernatant were loaded into the SDS-PAGE gel slots.

### Glucuronokinase activity assays

Detection of DrGK activity was performed using coupled HPLC enzyme assays. Enzyme assays were carried out in 0.2 ml reaction tubes at a final volume of 60 µl containing 50 mM MOPS-KOH (pH 7.5), 4 mM MgCl_2_, 1 mM ATP, 1 mM UTP, 1 mM GlcA, 1 µg recombinant UDP-sugar pyrophosphorylase [21] from *Pisum sativum* as coupling enzyme and 15 ng of either recombinant DrGKUP, DrGK *or* XtGKUP. Coupled HPLC enzyme assays were incubated in a PCR cycler at 35°C for 20 min and reactions were stopped by heating the tubes to 95°C for 5 min. For HPLC analysis, 20 µl of the enzyme assay were injected. Determination of biochemical data like pH and temperature optimum of the recombinant DrGK were performed by measurements of produced UDP-GlcA. Beside Mg^2+^ as cofactor, several other cations were tested as cofactors of DrGK. Therefore a standard kinase assay; in which Mg^2+^ was replaced by other cations, was heat inactivated and produced GlcA-1-phosphate was determined with recombinant UDP-sugar pyrophosphorylase (including Mg^2+^) from *Pisum sativum* as coupling enzyme. Control experiments were performed with Glc-1-phosphate and cations to ensure that the pea pyrophosphorylase is not blocked in the presence of the investigated cations.

### UDP-Glucuronic Acid Pyrophosphorylase Activity, Synthesis Direction

Detection of putative DrUP activity was performed using standard HPLC enzyme assays carried out in 0.2 ml reaction tubes with a final volume of 60 µl containing 50 mM MOPS-KOH (pH 7.5), 4 mM MgCl_2_, 1 mM ATP, 1 mM UTP, 1 mM GlcA and 75 ng of either recombinant DrGKUP, DrUP or XtGKUP. HPLC enzyme assays were incubated in a PCR cycler at 35°C for 20 min and reactions were stopped by heating the tubes to 95°C for 5 min and analysed on HPLC. In standard HPLC enzyme assay, *DrGKUP* was assumed to catalyze the reaction GlcA+ATP+UTP→UDP-GlcA+ADP+PPi. Though, detection of UDP-GlcA in standard HPLC enzyme assay was not successful we tested several different HPLC enzyme assays and possible reaction variations. We alternated the buffer systems with different pH values (potassium-acetate pH 4–5.5, MES-KOH pH 5–7, MOPS-KOH pH 6.5–8, Tris -HCl pH 7.5–9,5), the mono-, di- and trivalent cations (Mg^2+^, Mn^2+^, Ca^2+^, Co^2+^, Ba^2+^, K^+^, Li^+^, Al^3+^, Fe^3+^), the nucleotide triphosphates (ATP, CTP, GTP, TTP, dATP, dCTP, dGTP, dTTP), the ratio of Mg^2+^/UTP (between 5 and 10), supplemented enzyme stabilizing factors (0.5 mg/ml BSA, 3% Glycerin, 10% Glycerin, 1 mM DTT, 1 mM EDTA, 0.15% Triton X-100, 0,15% Tween20, 0,15% NP40, 1 mM Zn^2+^), added different sugar-1-phosphates than GlcA-1-phosphate as substrate (*Glc-1-phosphate*, *Gal-1-phosphate*) and supplied other UDP-sugars (*UDP-Glc*, *ADP-Glc*, *TDP-Glc*, *GDP-Glc*, *UDP-GlcNAc*) for possible transferase activities.

Long time enzyme assays were carried out in 0.2 ml reaction tubes at a final volume of 60 µl containing 50 mM MOPS-KOH (pH 7.5), 4 mM MgCl_2_, 1 mM ATP, 1 mM UTP, 1 mM GlcA, 0.15% Tween 20 and 15 ng of either recombinant DrGK, DrUP or DrGKUP. Control reactions were carried out in 0.2 ml reaction tubes at a final volume of 60 µl containing 50 mM MOPS-KOH (pH 7.5), 4 mM MgCl_2_, 1 mM ATP, 1 mM UTP, 1 mM GlcA, 0.15% Tween 20, 15 ng of recombinant DrGKUP and 1 µg recombinant UDP-sugar pyrophosphorylase from *Pisum sativum* as coupling enzyme with or without 1 µg inorganic pyrophosphatase from yeast (Roche). HPLC enzyme assays were incubated in a PCR cycler at 35°C for 70 hours and reactions were stopped by heating the tubes to 95°C for 5 min. For HPLC analysis, 20 µl of the enzyme assay were injected.

### UDP-Glucuronic Acid Pyrophosphorylase Activity, Pyrophosphorolysis Direction

Detection of putative DrUP activity was performed using standard HPLC enzyme assays. HPLC standard enzyme assays were carried out in 0.2 ml reaction tubes at a final volume of 60 µl containing 50 mM MOPS-KOH (pH 7.5), 4 mM MgCl_2_, 1 mM UDP-GlcA, 1 mM PP_i_ and 75 ng of either recombinant DrGKUP, DrUP or XtGKUP. HPLC enzyme assays were incubated in a PCR cycler at 35°C for 20 min and reactions were stopped by heating the tubes at 95°C for 5 min and analysed on HPLC.

### HPLC Detection of UDP-GlcA

UDP-GlcA produced during the standard and coupled HPLC enzyme assays as well as UDP-GlcA produced in metabolic pathway design experiments in *S. cerevisiae* cells were measured by a Dionex UltiMate 3000 Rapid Separation LC System (Thermo Scientific) using a NUCLEOSIL® 4000-7PEI (Macherey-Nagel) strong anion exchange (SAX) column cartridge (125×4.0 mm column size) for chromatography of nucleotides analyzing data with Chromeleon 7.12 (Thermo Scientific). Temperature of the column compartment was set at 25°C during analysis and separation of the different assay components was performed using the following eluents: A (2.5 mM Tris/phosphate (pH 7.2) and B (2.5 mM Tris/phosphate (pH 8.0)+1.5 M KCl), and the following gradient for standard and coupled HPLC enzyme assays: 5% B to 95% B in 5 min, flow rate 1.3 ml·min^−1^. Elution times of reference components were 2.06 min for UMP, 2.44 min for AMP, 2.87 min for UDP-GlcA, 3.85 min for UDP, 4.54 min for ADP, 5.28 min for UTP and 6.09 min for ATP. Separation of NDP-sugars from *S. cerevisiae* was performed using the following gradient: 5% B to 19.9% B in 5.8 min, 19.9% B to 21.9% B in 3.2 min and 21.9% B to 95% B in 1 min, flow rate 1.3 ml·min^−1^. Elution time of reference component was 5.96 min for UDP-GlcA. UV spectra were recorded between of 240 nm and 300 nm and the chromatogram was displayed for 260 nm.

### LC/MS Analysis of UDP-GlcA

UDP-GlcA produced during the 70 hours standard HPLC enzyme assays was measured by an Exactive bench-top system (Thermo Scientific) which features an Orbitrap mass analyzer. Standard HPLC enzyme assays were separated by an Accela LC system (Thermo Scientific) using a NUCLEODUR® C18 Gravity (Macherey-Nagel) high-purity silica phase column cartridge (250×3.0 mm column size, 5 µm particle size) for chromatography of nucleotides analyzing data with Chromeleon 7.1 Chromatography Data System (Thermo Scientific). Temperature of the column compartment was set at 25°C during analysis and separation of the different assay components was performed using the following eluents: A (2.5% acetonitrile in water with 0.1% formic acid, adjusted to pH 4 with ammonia) and B (50% acetonitrile in water with 0.1% formic acid, adjusted to pH 4 with ammonia), and the following gradient for standard HPLC enzyme assays: isocratic separation with 100% A for 15 min, flow rate 0.5 ml·min^−1^. Elution time of reference component was 1.9 min for UDP-GlcA. The LC-MS experiments were performed by coupling an Accela LC system to the Exactive MS system (Thermo Scientific). This was done by using fused silica capillaries with an inner diameter of 100 µm. The mass spectrometer was calibrated according to the manufacturer's instructions using an IonMAX ESI source. UDP-GlcA was detected in negative mode as deprotonated molecules. The tuning of the mass spectrometer was done by hyphenating the HPLC system to the IonMAX ESI source. The following scan parameters were used during measurements, Scan range: 100 to 2000 m/z, Resolution: Ultra High, Microscans: 1, AGC target: Balanced, Maximum inject time: 50 ms. Source settings had to be established with a spray voltage of 2 kV, capillary temperature of 250°C and sheath gas of 10 arb. Lock masses have been used for a formic acid dimer ([M_2_+Na-2H]^−^, m/z 112.98563) and trifluoroacetic acid dimer ([M_2_-H]^−^, m/z 226.97845), both of these masses were permanently present during the measurements.

## Results

### Sequence Analysis

**Figure 2:**
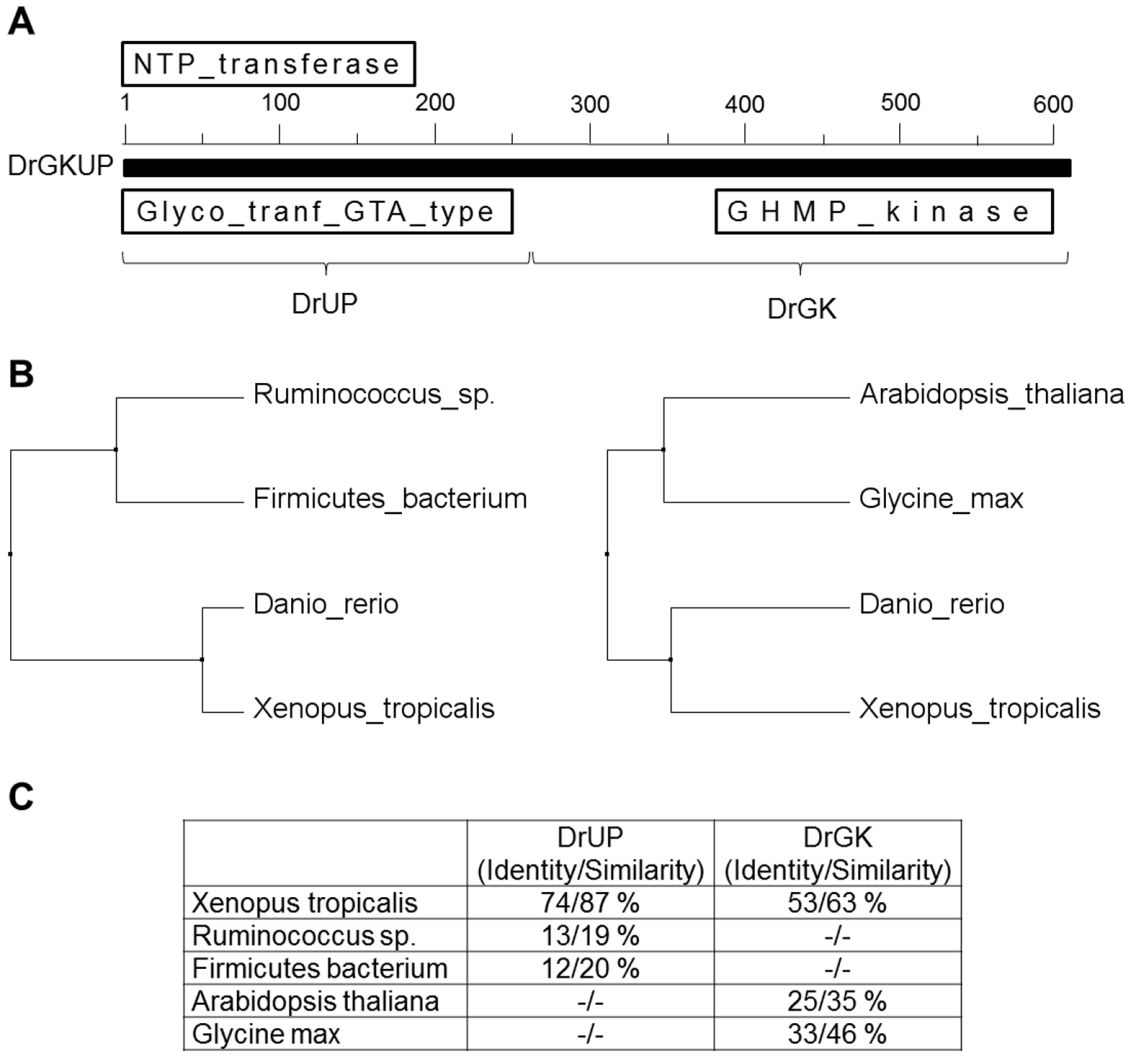
Sequence and Phylogenetic Analysis of DrGKUP. (A) shows a bifunctional enzyme comprised of a putative kinase domain (DrGK; 357 amino acids; 40 kDa) at the C-terminal end and a putative pyrophosphorylase domain (DrUP; 260 amino acids; 30 kDa) at the N-terminal end. For the pyrophosphorylase domain analysis *Danio rerio* (XP_005157584.1), *Xenopus tropicalis* (XP_002940431.1), *Ruminococcus_sp*. (WP_021925996.1) and *Firmicutes_bacterium* (WP_022231022.1) were aligned and for glucuronokinase domain analysis *Danio rerio* (NP_001107088.1), *Xenopus tropicalis* (AAI55522.1), *Arabidopsis thaliana* (AAV74231.1) and *Glycine max* (NP_001242154.1) were aligned and with the pairwise method using Clustal Omega. (C) Identity and Similarity were calculated with GENEDOC and (B) phylogenetic distance trees were calculated with JalView. Neither a homologous glucuronokinase nor a pyrophosphorylase sequence could be found in higher animals or humans.

**Figure 3:**
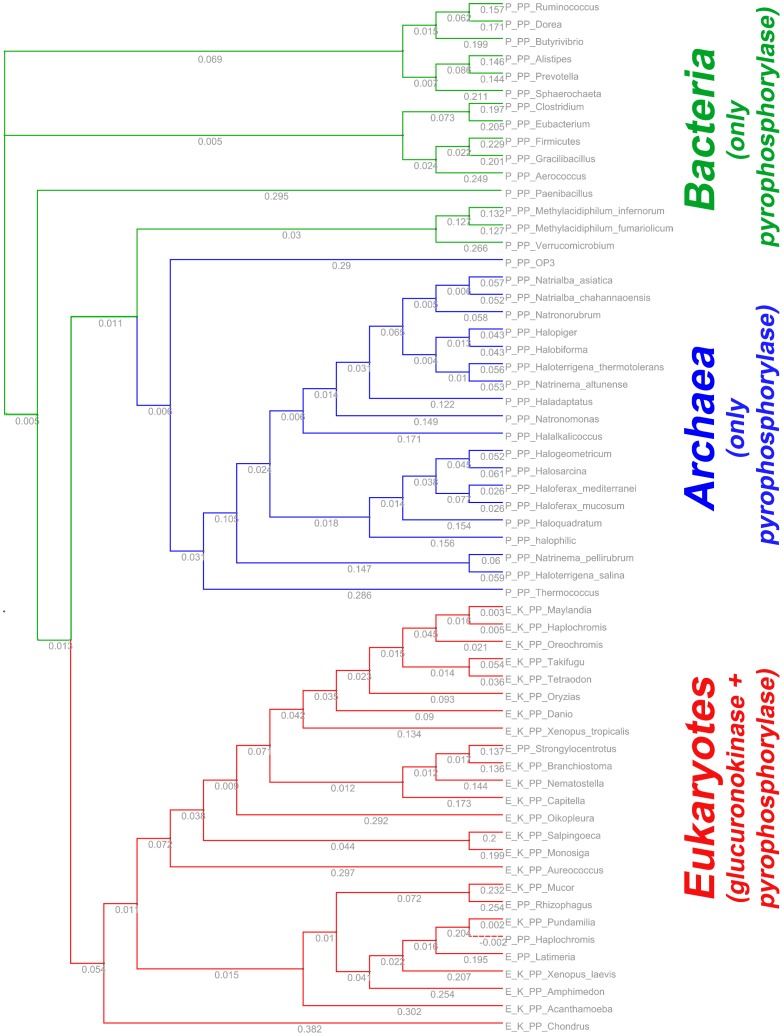
Distance tree of one group of pyrophosphorylases from pro- and eukaryotes calculated from an alignment with CLUSTAL. In bacteria and archaea the proteins only encode a pyrophosphorylase whereas in eukaryotes a fusion protein with glucuronokinase in typically found. For the latter group only the pyrophosphorylase domain was used in the alignment. Each species name has a prefix (E: eukaryote; P: prokaryote) followed by the type of protein (PP: only pyrophosphorylase; K_PP: glucuronokinase and pyrophosphorylase). The fusion proteins are found e.g. in fungi, tunicates, polychataea, amphibian and fish. *Danio rerio* (gi|528501576), *Oreochromis niloticus* (gi|542261080), *Maylandia zebra* (gi|498923801), *Haplochromis burtoni* (gi|554854526), *Oryzias latipes* (gi|432895574), *Takifugu rubripes* (gi|410910724), *Xenopus tropicalis* (gi|301622206), *Strongylocentrotus purpuratus* (gi|390355576), *Branchiostoma floridae* (gi|260786665), *Nematostella vectensis* (gi|156391024), *Capitella teleta* (gi|443696256), *Salpingoeca rosetta* (gi|514691982), *Tetraodon nigroviridis* (gi|47224901), *Monosiga brevicollis* MX1 (gi|167517681), *Aureococcus anophagefferens* (gi|323454734), *Oikopleura dioica* (gi|313221557), *Acanthamoeba castellanii* (gi|470509640), *Mucor circinelloides* (gi|510999707), *Xenopus laevis* (gi|163914505), *Amphimedon queenslandica* (gi|340375174), *Ruminococcus sp.* (gi|547183951), *Latimeria chalumnae* (gi|556979378), *Firmicutes bacterium* (gi|547822613), *Pundamilia nyererei* (gi|548393190), *Clostridium sp.* (gi|547295929), *Gracilibacillus lacisalsi* (gi|517760452), *Eubacterium sp.* CAG:274 (gi|547841474), *Methylacidiphilum infernorum* V4 (gi|189220259), *Methylacidiphilum fumariolicum* (gi|496352483), *Rhizophagus irregularis* (gi|552908620), *Natrialba asiatica* (gi|493050309), *Natronorubrum bangense* (gi|492959802), *Paenibacillus sp.* (gi|518757082), OP3 bacterium SCGC (gi|551125677), *Halalkalicoccus jeotgali* (gi|300711896), *Natrinema pellirubrum* (gi|433593563), *Natrialba chahannaoensis* (gi|493163024), *Aerococcus viridans* (gi|515407717), *Alistipes sp.* (gi|546342953), *Halogeometricum borinquense* (gi|313127001), *Haloferax mediterranei* (gi|389846545), *Sphaerochaeta globosa* (gi|325970222), *Halopiger xanaduensis* (gi|336255356), *Prevotella sp.* (gi|547906812), *Natronomonas pharaonis* (gi|76800846), *Haloferax mucosum* (gi|495596680), *Haloterrigena thermotolerans* (gi|493700315), *Haloterrigena salina* (gi|496171985), *Halosarcina pallida* (gi|495664926), *Butyrivibrio proteoclasticus* (gi|302671768), *Haplochromis burtoni* (gi|554859531), *Natrinema altunense* (gi|494168028), *Thermococcus litoralis* (gi|530548455), *Halophilic archaeon* DL31 (gi|345004592), *Dorea formicigenerans* (gi|547873312), *Halobiforma nitratireducens* (gi|493722014), *Chondrus crispus* (gi|546322381), *Haloquadratum walsbyi* (gi|544617810), *Verrucomicrobium sp*. (gi|521982779) and *Haladaptatus paucihalophilus* (gi|495256835).

A database search using the glucuronokinase sequence from Arabidopsis clearly revealed homologous sequences in zebrafish. The C-terminal part of the protein sequence displays typical motifs for GHMP-kinases, a small group of metabolite kinases which includes all known sugar-1-kinases so far. Surprisingly, the zebrafish predicted protein has a long (∼260 amino acids) N-terminal domain, which is absent from plants. This domain shows sequence similarity to nucleotidyltransferases from bacteria within the Glycosyl-GTA-type superfamily (Fig. 2). BLAST homology searches in GenBank™ revealed no other homologs in zebrafish. Highly similar sequences to DrGKUP were found in some basal animals (amphibians, fishes, cnidarians, tunicates) but not in mammals. We therefore performed a homology search using only the predicted nucleotidyltransferase domain and aligned sequences with CLUSTAL. Three branches are clearly separated, representing eubacteria, archaea and eukaryotes (Fig. 3). Whereas the nucleotidyltransferase sequences from eubacteria and archaea are only comprised of this domain, the eukaryotic sequences are in general fusion proteins with glucuronokinase. We found two exceptions in GenBank (*Latimeria chalumnae*; *Strongylocentrotus purpuratus*), in which both domains seem to be encoded by two independent transcripts.

**Figure 4:**
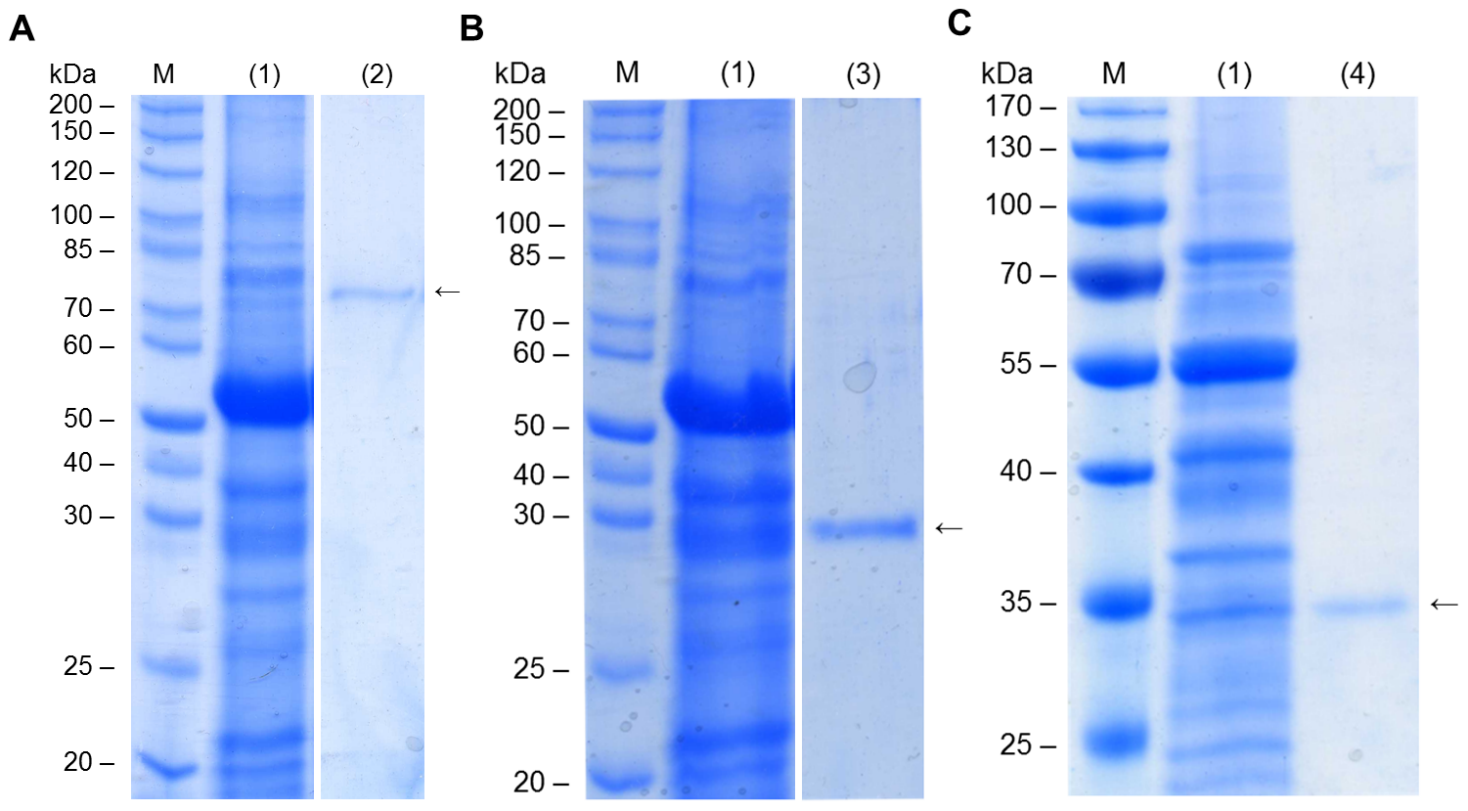
SDS-PAGE of DrGKUP Purification Expressed in *N. benthamiana*. Tobacco leaf crude extract (A, 1) and purified recombinant DrGKUP (A, 2). Crude extract (B, 1) and purified recombinant DrGK (B, 3) and crude extract (C, 1) and purified recombinant DrUP (C, 4).

To confirm the function of the identified protein we expressed the full-length open reading frame of DrGKUP, encoding 619 amino acids with a molecular mass of about 70 kDa in *E. coli* using the pET-32c vector with His_6_-tag. In BL21 cells, all of the protein was found in inclusion bodies under various conditions. Using *E. coli* Origami cells we obtained only a small amount of soluble protein that has glucuronokinase activity. We therefore switched to a eukaryotic expression system. Expression in tobacco leaves was achieved by transient infiltration of *Agrobacterium tumefaciens* with a suitable pGreen-based expression vector. Instead of a His_6_-tag the plant expression vector allows a fusion with the StrepII-tag allowing purification of the recombinant full length protein (DrGKUP) under mild conditions (Fig. 4). As the bioinformatics already suggested a bifunctional enzyme comprised of a putative kinase domain (DrGK; 357 amino acids; 40 kDa) and a putative pyrophosphorylase domain (DrUP; 260 amino acids; 30 kDa) we also expressed the domain individually in tobacco leaves (Fig. 4).

### HPLC Enzyme Assay Design

**Figure 5:**
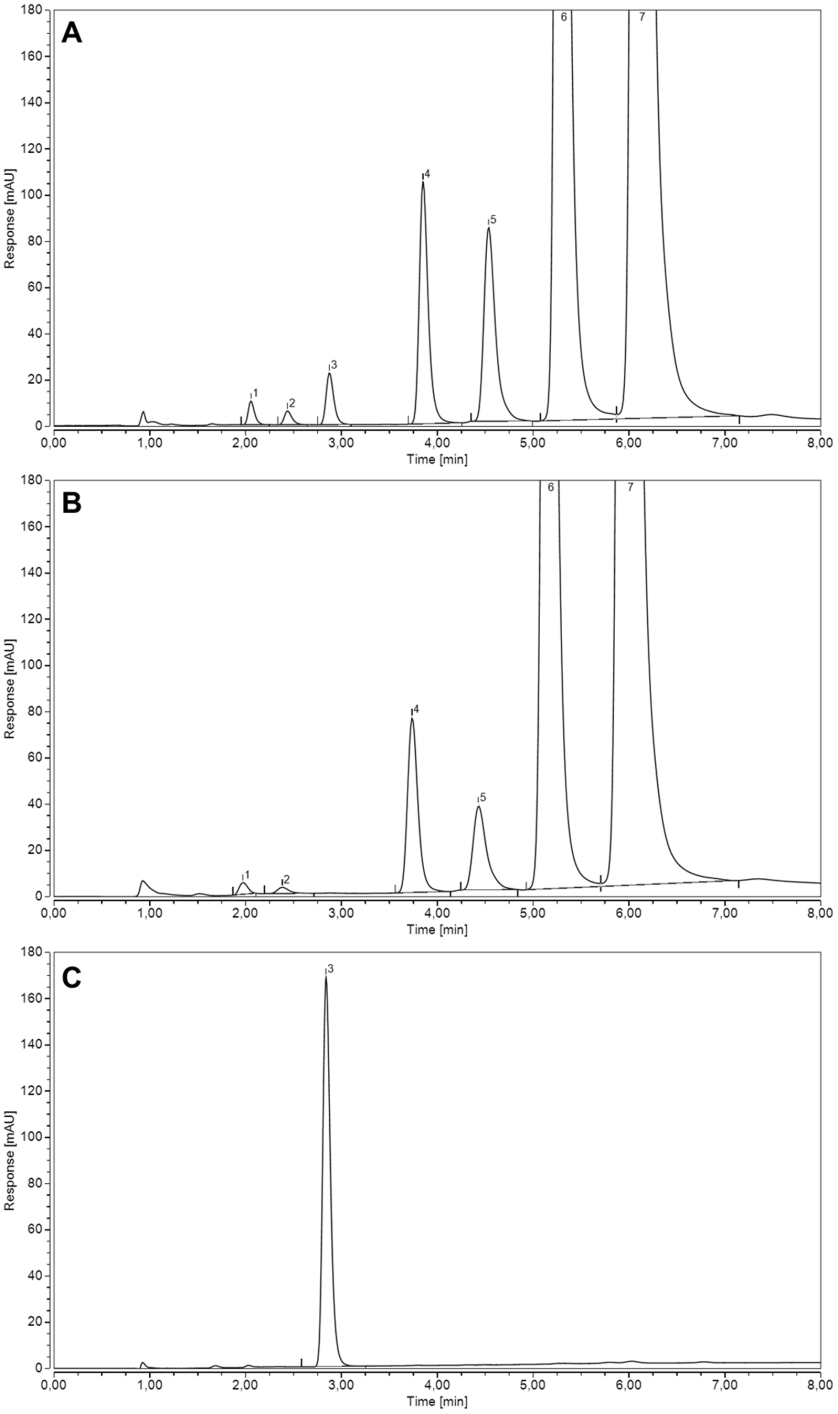
Coupled HPLC Enzyme Assay of DrGK. (A) Enzyme activity of DrGK was tested. Peak (3) indicates produced UDP-GlcA during coupled enzyme reaction. Peak (1) UMP, (2) AMP, (4) UDP, (5) ADP, (6) UTP and (7) ATP. (B) Control without DrGKUP. (C) 50 µM UDP-GlcA as reference compound.

The recombinant full length DrGKUP did not convert GlcA into UDP-GlcA in short period of time enzyme assays using conditions which general support activities of the kinase and the pyrophosphorylase. These conditions include neutral buffer with MgCl_2_, ATP, UTP and GlcA as a substrate. Therefore we decided to measure the activity of the different domains individually. We first started by characterizing the glucuronokinase domain. The product GlcA-1-phosphate is not readily detectable in HPLC. Therefore we made use of a stable UDP-sugar pyrophosphorylase from pea [21] as a coupling enzyme, which can easily be expressed as a recombinant enzyme in *E. coli*. This enzyme has been shown to readily convert GlcA-1-phosphate into UDP-GlcA in the presence of UTP [30]. The product of the enzyme assay was finally analysed on HPLC and detected by UV absorption (262 nm). A typical chromatogram (Fig. 5A) with appropriate controls is shown in Fig. 4B. The recombinant full length DrGKUP as well as the DrGK alone catalyzes the phosphorylation of GlcA in the coupled HPLC enzyme assay. The product is dependent on the presence of ATP, UTP and GlcA and identical with the commercially available reference compound UDP-GlcA (Fig. 5C). Concomitant, the intermediate ADP accumulate linearly with UDP-GlcA product formation. These experiments confirm that the purified enzyme indeed has glucuronokinase activity. Characterization of DrGK was performed in enzyme assays not exceeding 20 min, in which the amount of UDP-GlcA increased linear with reaction time. At later time points the product accumulation drops, indicating that either product inhibition by ADP, PP_i_ or GlcA-1-phosphate occurs or that DrGK is not stable and therefore enzyme activity decreases. Product inhibition was excluded experimentally by adding ADP, PP_i_ and GlcA-1-phosphate in excess which did not decrease enzyme activity. To increase DrGK stability we added different stabilizing factors to the coupled HPLC enzyme assay proceeding for 4 hours (Fig. S1). The addition of 0.15% Tween20 increased UDP-GlcA product formation by 3-fold compared to controls without any stabilizing factor.

### Glucuronokinase Characterization

**Figure 6:**
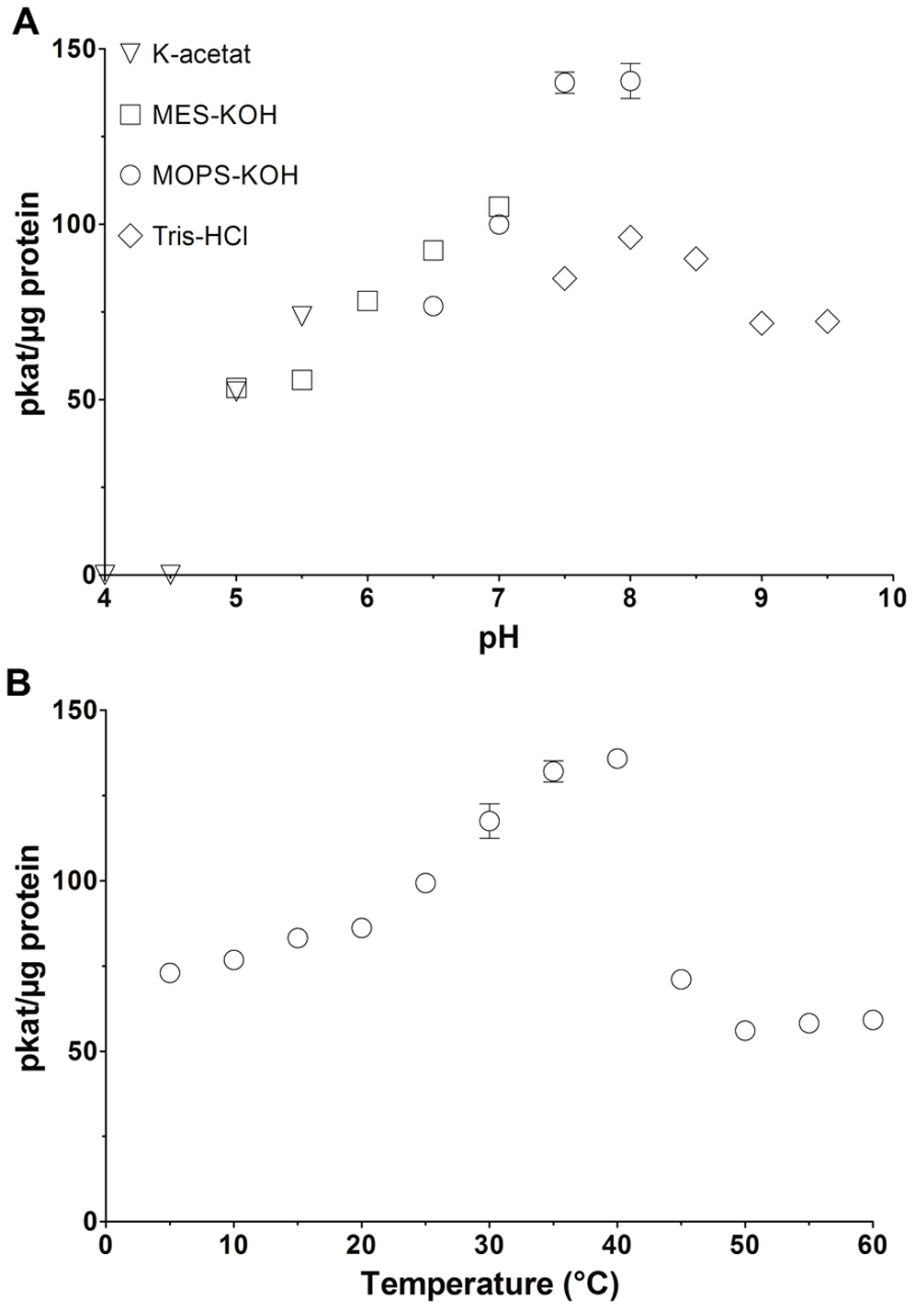
pH-Optimum and Temperature-Optimum of DrGK. (A) The effect of different pH values on DrGK activity was measured with coupled HPLC enzyme assay at different pH values dependent on the used buffers (50 mM). ▪potassium-Acetat: pH 4–5; ♦MES-KOH: pH 5.5–6.5; ▴MOPS-KOH: pH 7–7.5; •Tris-HCl: pH 8–9.5. UDP-GlcA level was determined using coupled HPLC enzyme assay. Values are averages of three independently performed assays (+/− SD). (B) The dependence of DrGK activity on reaction temperature was measured with coupled HPLC enzyme assay at different temperatures within a range from 10°C to 60°C. UDP-GlcA level was determined using coupled HPLC enzyme assay. Values are averages of three independently performed assays (+/− SD).

Activity of DrGK was determined at different pH values ranging from pH 4.0 to 9.5 (Fig. 6A), using UDP-GlcA formation as the detection method. No activity was measured below pH 4.5, whereas the maximum was reached between pH 7.5 and 8 with a far lower activity above pH 8.5. Utilization of Tris-Cl buffer, pH 7.5, under standard conditions showed a 40% decrease of enzyme activity compared with MOPS-KOH buffer at the same pH. DrGK showed enzyme activity within a temperature range between 10 and 60°C (Fig. 6B). The optimum was located between 35 and 40°C. Temperatures above 45°C lead to rapid inactivation of the enzyme. For most kinases divalent cations are necessary for substrate conversion. The effect of different monovalent, divalent, and trivalent metal ions was tested (Tab. 1) and UDP-GlcA production was monitored during coupled enzyme assay by HPLC measurement. The corresponding ions (1 mM) were applied and values were calculated referenced to the magnesia used as the divalent cation in the coupled HPLC enzyme assay. DrGK requires Mg^2+^ but also other divalent cations, like Mn^2+^ and Co^2+^ were able to substitute for magnesium. None substrate turnover was measured neither with monovalent nor with trivalent cations. As the assay involves recombinant UDP-sugar pyrophosphorylase from pea, controls with glucose-1-phosphate as a substrate were performed ensuring that the enzyme is active in the presence of the tested metal ions.

**Table 1:** Metal Ion Requirement of DrGK for Phosphorylation of GlcA.

Metal Ions [1 mM]	Relative Activity [%]
Mg^2+^	100.0±1.5
Mn^2+^	78.5±1.5
Co^2+^	62.8±0.7
Ca^2+^	0.0±0.0
Ba^2+^	0.0±0.0
K^+^	0.0±0.0
Li^+^	0.0±0.0
Al^3+^	0.0±0.0
Fe^3+^	0.0±0.0

In a standard assay, Mg^2+^ was exchanged for other cations and the enzyme activity is reported.

### Substrate Specificity

**Figure 7:**
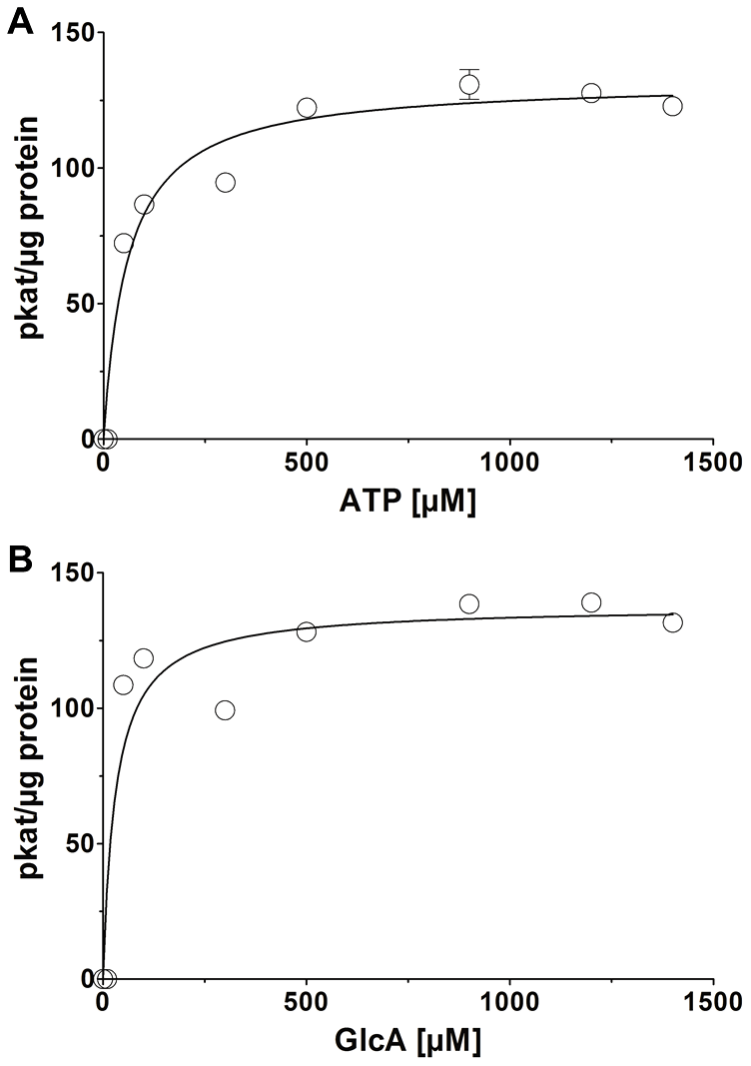
Kinetics of DrGK for ATP and GlcA. (A) Substrate saturation by Michaelis-Menten curve for ATP is shown. Activity of DrGK was measured with varying concentrations of ATP (0.01–1.4 mM) under standard conditions for 20 min with coupled HPLC enzyme assay. K_m_ with 59.3 µM and V_max_ 132.1 pkat/µg protein was calculated. Values are averages of three independently performed assays (+/− SD). (B) Substrate saturation by Michaelis-Menten curve for GlcA is shown. Activity of DrGK was measured with varying concentrations of *GlcA* (0.01–1.4 mM) under standard conditions for 20 min with coupled HPLC enzyme assay. K_m_ with 31.3 µM and V_max_ 137.6 pkat/µg protein was calculated. Values are averages of three independently performed assays (+/− SD).

To determine the monosaccharide substrate specificity for the kinase reaction standard enzyme assays with ATP and different sugar substrates were performed. Monosaccharides (1 mM) like D-glucuronic acid, D-glucose, D-xylose, L-arabinose, L-fucose, D-mannose, L-rhamnose, D-galacose and D-galacturonic acid were used and both, the increase of ADP due to ATP consumption during the kinase reaction and UDP-sugar formation were measured (Tab. 2). DrGK only catalyzed the conversion of GlcA into its corresponding sugar-1-phosphate but failed to utilize any other monosaccharide tested in the coupled HPLC enzyme assays, indicating a very high substrate specificity of DrGK for the sugar GlcA. We also tested different nucleotide triphosphates as phosphate donors in the kinase reaction. For this we used 1 mM of ATP, CTP, GTP, TTP, dATP, dCTP, dGTP and dTTP (Tab. 3). Apart from ATP none of the nucleotides was able to serve as a phosphate donor for glucuronokinase. Kinetic analyses were performed for the substrates ATP and GlcA (Tab. 4). The enzyme kinetic for ATP shows a hyperbolic curve from which a K_m_ value for ATP of 59±9.1 µM, a V_max_ value of 132.1 pkat/µg protein and a k_cat_ value of 1.3 s^−1^ was calculated (Fig. 7A). Substrate saturation curves for GlcA followed a hyperbolic curve according to a Michaelis-Menten kinetic (Fig. 7B). The K_m_ value of the enzyme for GlcA in a substrate range of 0.01 to 1.4 mM was calculated as 31±8.0 µM, as well as a V_max_ value of 137.6 pkat/µg protein and a k_cat_ value of 1.4 s^−1^.

**Table 2:** Monosaccharide Substrate Specificity of DrGK.

Substrate [1 mM]	Relative Activity [%]
D-glucuronic acid	100.0±2.8
D-glucose	ND
D-xylose	ND
L-arabinose	ND
L-fucose	ND
D-mannose	ND
L-rhamnose	ND
D-galactose	ND
D-galacturonic acid	ND

In a standard enzyme assay the substrate glucuronic acid was exchanged for other common sugars. ND: not detectable.

**Table 3:** Nucleotide Triphosphate Substrate Specificity of DrGK.

Substrate [1 mM]	Relative Activity [%]
UTP+ATP	100.0±1.4
UTP	0.0±0.0
UTP+GTP	0.0±0.0
UTP+CTP	0.0±0.0
UTP+TTP	0.0±0.0

**Table 4:** Enzyme Kinetics of DrGK.

	K_m_ (sugar) [µM]	K_m_ (NTP) [µM]
DrGK	31.3±8.0	59.3±9.1
AtGalAK	70.8	195
ScGalK	600	150
HsGalK	970	34
AtGlcAK1	697	555
AtGalK	701	701
AtFK	1000	450

Comparision of kinetic parameters of different sugar-1-kinases. Arabidopsis galacturonic acid kinase AtGalAK [20]; human galactokinase HsGalK [49], yeast galactokinase ScGalK [50], Arabidopsis glucuronokinase AtGlcAK [16], Arabidopsis galactokinase AtGalK [18], Arabidopsis fucokinase AtFK [19].

### UDP-Glucuronic Acid Pyrophosphorylase Activity

**Figure 8:**
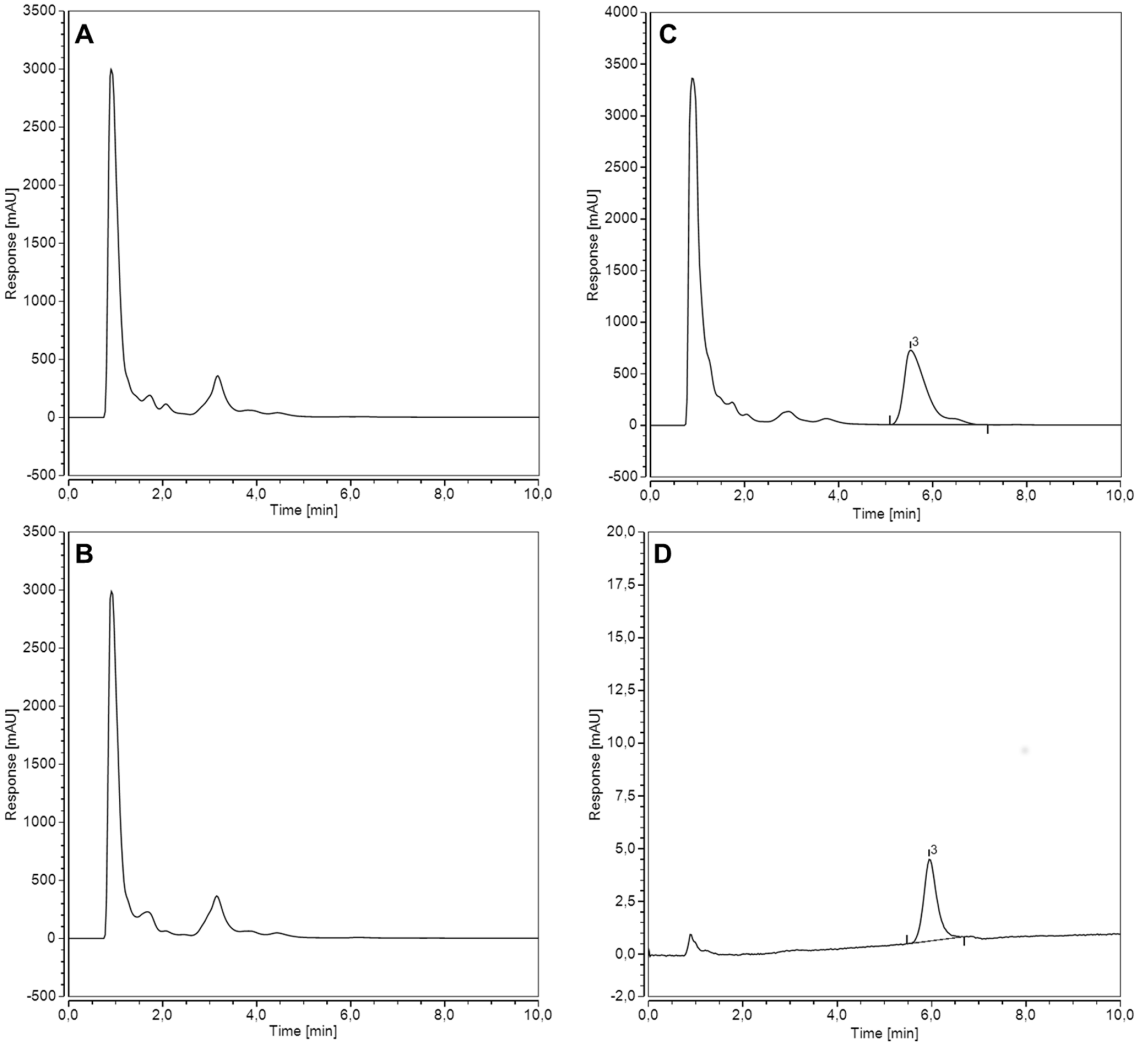
Metabolic pathway design experiments in *S. cerevisiae* cells. Metabolite profiles from yeast expressing recombinant proteins. (A) DrGKUP, (B) empty pToy4 vector control, (C) pToy4 expressing Arabidopsis UDP-glucose dehydrogenase 1 (D) 50 µM UDP-GlcA as reference compound.

Because no DrUP activity could be detected in standard HPLC enzyme assay, we tested different buffer systems and pH values, various mono-, di- and trivalent cations, and eight different nucleotide triphosphates. In addition, the ratio of Mg^2+^/UTP was varied and enzyme stabilizing factors were added. We also added pyrophosphatase to the enzyme assay to drive a possible equilibrium between sugar-1-phosphate and NTP to NDP-sugar and pyrophosphate by hydrolysing the latter compound. The putative pyrophosphorylase was incubated with different nucleotide-triphosphates alone but did not hydrolyse them into nucleotide-monophosphates and pyrophosphate (data not shown). In many UDP-sugar pyrophosphorylase enzymes (mouse, *Leishmania*, barley, with D-glucose as substrate) a sequential ordered mechanism is reported in which UTP binds first and induced fit of this binding allows the sugar phosphate to bind. We therefore purified DrUP or DRGKUP in the presence of 100 µM UTP and ATP to potentially stabilize the enzyme. Again the enzyme purification in the presence of these cofactors does not increase the pyrophosphorylase activity. To ensure, that the DrUP has available all co-factors of typical eukaryotic cells to perform pyrophosphorylase activity, we designed a metabolic pathway experiment in *S. cerevisiae*, since we had evidence that *S. cerevisiae* could uptake GlcA [31], [32]. Previously, Oka und Jigami [25] had expressed a plant UDP-glucose dehydrogenase in yeast and showed that UDP-GlcA accumulates as stable product in these cells. We were able to repeat and confirm this experiment (Fig. 8). Nevertheless, the expression of DrGKUP in yeast and feeding of GlcA does not lead to an accumulation of UDP-GlcA, indicating that metabolites of yeast cells are not required for the DrGKUP enzyme.

**Figure 9:**
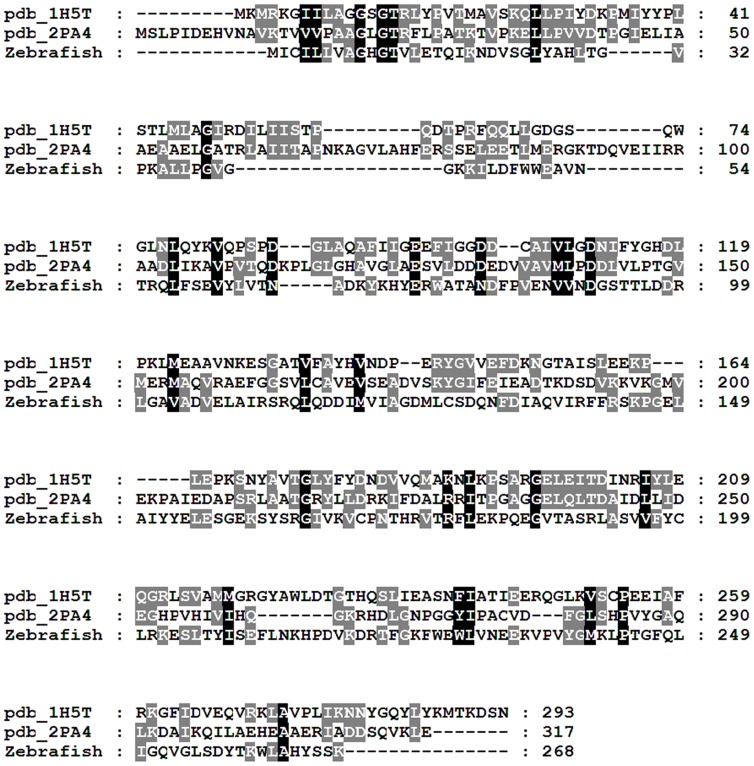
Sequence alignment of two bacterial nucleotidyltransferase with known crystal structure (1H5T [33]; 2PA4 [51]) and the pyrophosphorylase domain from zebrafish DrGKUP. Note that some of the residues identified in the *E. coli* enzyme to bind the substrate are conserved in the zebrafish sequence (e.g. Gly11; Asp91; Asp118; Lys163; Arg220; numbers refer to the *E. coli* sequence 1H5T).

Next we considered the possibility that the gene coding for DrGKUP might have critical mutations in the pyrophosphorylase domain, rendering the enzyme non-functional. We compared the zebrafish enzyme with homologous nucleotidyltransferases from bacteria for which protein structures are known. The sequences are co-linear and share conserved residues with the zebrafish enzyme (Fig. 9). Some of the amino acids involved in substrate binding in the *E. coli* glucose-1-thymidylyltransferase [33] are conserved in the zebrafish enzyme (e.g. Gly11; Asp91; Asp118; Lys163; Arg220 number refer to the *E. coli* sequence).

**Figure 10:**
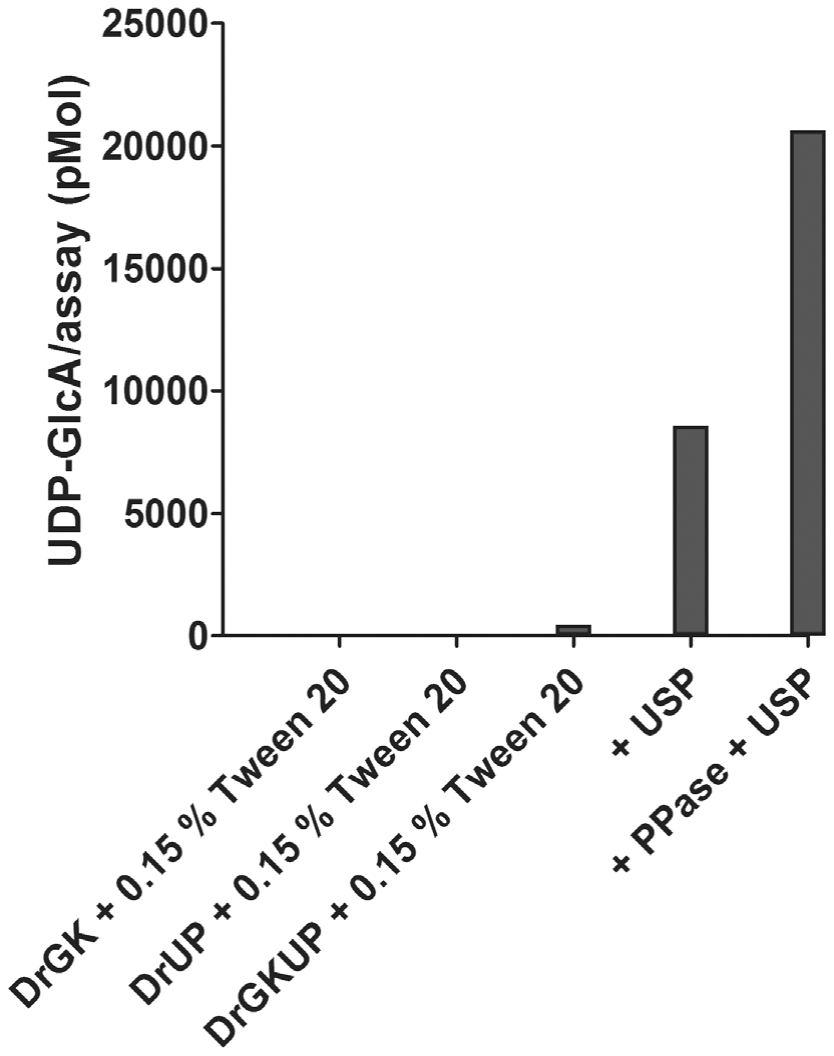
Long time Enzyme Assay. We performed a long time enzyme assay for 70% Tween20 as enzyme stabilizing factor. Under these conditions, we could detect a small amount of UDP-GlcA signal on HPLC. Two control assays with either DrGK or DrUP alone did not result in any detectable product formation. For comparison and as a positive control, two enzyme assays are shown, in which the recombinant plant pyrophosphorylase (USP) was added.

To exclude the scenario of inactivity due to mutation of critical residues, we expressed the homologous enzyme from *Xenopus tropicalis* as a recombinant protein in tobacco. Again we can confirm the glucuronokinase activity for the *Xenopus* enzyme but were unsuccessful for the corresponding putative pyrophosphorylase activity in standard HPLC enzyme assays. Since DrGK characterization provided data for the highest enzyme activity at a pH of 7.5 in MOPS-KOH buffer, a temperature of 35°C, magnesia as divalent cation, ATP as phosphate donor and GlcA as substrate, we performed a long time enzyme assay for 70 hours (Fig. 10) adding additionally UTP as nucleotide diphosphate donor for DrGKUP and 0.15% Tween20 as enzyme stabilizing factor. Under these conditions, we could detect a small amount of UDP-GlcA signal on HPLC, which is identical with the commercially available reference compound UDP-GlcA and exhibits a UV spectrum with 262 nm maximum as typically seen for uridine containing compounds. We also performed a LC/MS measurement of the product from the enzyme assay and confirmed that the mass of the product signal (m/z = 579.03) is identical to commercial UDP-GlcA reference compound (Fig. S2). As a positive control we added recombinant pea UDP-sugar pyrophosphorylase to the enzyme assay with DrGKUP, which results in UDP-GlcA formation. The addition of inorganic pyrophosphatase can even enhance the formation of UDP-GlcA by shifting the equilibrium to the nucleotide sugar biosynthesis direction. Controls with DrGK or DrUP did not show any signal for the product UDP-GlcA, suggesting that both proteins are needed for the (low) two step activity from GlcA to UDP-GlcA.

## Discussion

Here we describe the molecular cloning and biochemical characterization of a glucuronokinase with putative UDP-glucuronic acid pyrophosphorylase activitiy from zebrafish. The enzyme is clearly bifunctional and contains a glucuronokinase domain at the C-terminus and a putative pyrophosphorylase domain in the N-terminal part. As with other bifunctional sugar-1-kinase/pyrophosphorylases, the domains retain their properties when expressed separately as recombinant proteins. The glucuronokinase is a member of the GHMP (galacto-, homoserine, mevalonate, and phosphomevalonate) kinases superfamily. A common feature of them is a low sequence identity, but they all share similarities in their three-dimensional structure [34], [35], [36].). The sequence identity between the glucuronokinase from zebrafish and plants is surprisingly high considering the long evolutionary separation of plant and animal lineages. Therefore the often assumed evolution models for GHMP kinases – frequent exchanges of amino acids while maintaining the structure – is challenged by DrGK. The sequence identity/similarity of glucuronokinase between plant and animal sequences are higher than between other members of the GHMP kinase family within plants. An example for this is galacturonic acid-1-kinase from Arabidopsis [20], which takes a highly similar substrate to glucuronic acid but is very diverse in amino acid sequence.

DrGK is highly selective for the substrate glucuronic acid and accepts no other common sugar. DrGK exhibits a very high affinity towards GlcA (K_m_: 31±8 µM) compared to the homologous enzyme from Arabidopsis (K_m_: 697 µM). Other sugar-1-kinases of the GHMP kinase family often also display rather low affinities. The fucose kinase from Arabidopsis has a K_m_ of 1 mM [19] and yeast galactokinase binds to galactose with a K_m_ of 0.6 mM. In contrast, galacturonic acid kinase from Arabidopsis exhibits a high affinity binding (K_m_: 71 µM) [20]. What could be a reason for high affinity binding of GlcA to DrGK? The substrate GlcA is a precursor for ascorbic acid in animals. Use of GlcA for possible UDP-GlcA biosynthesis might compete as only limited information is known yet from both pathways.

The ability of animals to synthesize their own ascorbic acid got frequently lost during evolution without negative consequence as long as food provides enough ascorbic acid. Usually the genes for AsA biosynthesis are still present in animal genomes but mostly the terminal enzyme L-gulonolactone oxidase is non-functional because the gene turned into a pseudogene [37]. The gene for the bifunctional glucuronokinase and putative pyrophosphorylase however, seems to be totally absent from higher animal genomes. We are tempting to speculate that the competition for the substrate GlcA was avoided by elimination of the pathway from GlcA to UDP-GlcA giving the pathway from GlcA into ascorbic acid a clear preference.

For a bifunctional enzyme we would expect to find similar enzymatic activities for both, the kinase and the putative pyrophosphorylase reactions. The putative pyrophosphorylase activity of DrGKUP or the subunit DrUP is extremely low under our test conditions. We first tried standard conditions for pyrophosphorylases (neutral pH buffer, Mg^2+^, UTP and pyrophosphatase) which are used by most if not all pyrophosphorylases which have been characterized so far. These conditions work for enzymes from bacteria, animals and plants but not for the enzyme from zebrafish. [38] characterized a pyrophosphorylase from *Entamoeba histolytica*, which is totally inactive in an oxidized form but regains full activity in the presence of DTT or cysteine. This approach was not successful for DrGKUP. We then envisioned that an unknown cellular cofactor might be needed for pyrophosphorylase activity and expressed the enzyme in yeast cells to provide a larger range of potential cofactors. Again we were not successful to reconstitute the full enzyme activity of DrGKUP in yeast cells. Furthermore, ADP-glucose pyrophosphorylase is typically activated by metabolites like phosphoenolpyruvate, ribose-5-phosphate, Glc-6-phosphate or fructose-6-phosphate [39]. Addition of these metabolites (2 mM) to enzyme assays for DrGKUP did not increase the low pyrophosphorylase activity. This finding is not surprising and has been reported for other UDP-sugar pyrophosphorylases [39]. We next considered the possibility that the putative pyrophosphorylase domain is a pseudogene and therefore almost inactive. This hypothesis is far more difficult to test as a positive control (fully functional homologous enzyme) is missing. We took the homologous enzyme from *Xenopus tropicalis* (pyrophosphorylase shows 87% and glucuronokinase 63% sequence similarity) and could confirm the activity for the glucuronokinase but not for the pyrophosphorylase activity. Both sequences have no stop codon in the putative pyrophosphorylase domain and their sequences are co-linear without any striking insertions or deletions. This does not fully rule out the possibility of DrGKUP being a pseudogene but makes it rather unlikely. The structures of two bacterial nucleotidyl-transferases with homology to the putative pyrophosphorylase domain from zebrafish are available in the PDB database. The overall identity between the bacterial and the zebrafish enzymes is rather low. Nevertheless the sequences are co-linear and share some conserved residues including amino acids involved in substrate binding. Unfortunately both bacterial enzyme structures do not allow unambiguously the identification of the residues of the catalytic centre, which are clearly most important to be conserved. The great overall homology of the putative pyrophosphorylase domain in lower eukaryotes, which are all without internal stop-codons ranging from fungi (*Mucor circinelloides*) over tunicates to fish (many species) strongly suggest the maintenance of a functional pyrophosphorylase. These organisms separated about 650 million years ago [40]. In addition, no other pathway is known which could use GlcA-1-phosphate as a substrate. Without a suitable pyrophosphorylase, the activity of glucuronokinase is useless and just a waste of energy.

It seems thus possible that a posttranslational modification of DrGKUP is needed that is not attached to the protein in our expression systems, yeast and plant cells. Alternatively the DrGKUP protein needs another unidentified protein as a cofactor which boosts the enzymatic activity of the putative pyrophosphorylase domain. To our knowledge, examples for such a regulation of pyrophosphorylases are presently unknown.

In the human pathway of GAG and PG biosynthesis a gene-phenotype relationship is known for DFNA5 (hereditary nonsyndromic hearing impairment) and for CHSY1 (temtamy preaxial brachydactylyl syndrome). *UGDH* is a candidate gene, which is essential for embryonic development in general in frogs [41], fruit flies [42], nematodes [43] and mice [10], [44] and in particular in zebrafish where UGDH is critical for correct heart valve formation [8]. A quarter of all congenital heart diseases are caused by improper heart valve or heart septum morphogenesis [45] and hearing loss is the most common sensory disability in humans, about 1 of 500 new-borns has a congenital hearing loss [11], [46], [47], [48]. Given this variety and severity of human diseases, which are associated with this pathway, it is of indispensable importance to find an ideal model organism, like zebrafish, and explain any molecular or enzymatic differences to draw meaningful and correct conclusions for clinical basic research.

The main pathway for providing UDP-GlcA as precursor for HA involves the enzyme UGDH catalyzing the conversion of UDP-Glc to UDP-GlcA. The existence of a salvage pathway for GlcA to UDP-GlcA in lower vertebrates provides additional UDP-GlcA for GAG and PG biosynthesis. *jekyll/ugdh* zebrafish mutant phenotype is characterized by a very weak cartilage staining with Alcian blue. The UDP-GlcA salvage pathway catalysed by DrGKUP could be a possible explanation for the increase in Alcian blue staining in the pharyngeal cartilages in *jekyll/ugdh* zebrafish mutants that is stronger than that of *uxs1* zebrafish mutants [1], [8]. Isolation of zebrafish mutants in glucuronokinase, pyrophosphorylase and UDP-glucose dehydrogenase will help to elucidate the importance of glucuronokinase and pyrophosphorylase for the early organogenesis stages especially in skeletal including craniofacial and semicircular canal and coronary development.

## Supporting Information

Figure S1 **Stabilizing Factors.** The addition of stabilizing factors (0.5 mg/ml BSA, 3% Glycerin, 10% Glycerin, 1 mM DTT, 1 mM EDTA, 0.15% Triton X-100, 0,15% Tween20, 0,15% NP40, 1 mM Zn^2+^) increase DrGK activity in a coupled HPLC enzyme assay with 4 hours incubation time.
(TIF)

Figure S2 **LC/MS Measurement.** We also performed a LC/MS measurement of the UDP-GlcA produced during the 70 hours standard HPLC enzyme assays and confirmed that the mass of the product signal (m/z = 579.03) is identical to commercial UDP-GlcA reference compound. (A) 70 hours standard HPLC enzyme assay measurement (B) 50 µM UDP-GlcA as reference compound.
(TIF)

Table S1 **Primer sequences used to make expression constructs for the different protein domains.**
(DOCX)
