# Supplementary figures and images for: Molecular Cloning of a Novel Glucuronokinase/Putative Pyrophosphorylase from Zebrafish Acting in an UDP-Glucuronic Acid Salvage Pathway
Source: PLoS One. 2014 Feb 28;9(2):e89690. doi: 10.1371/journal.pone.0089690 (PMC3938481; doi:10.1371/journal.pone.0089690)

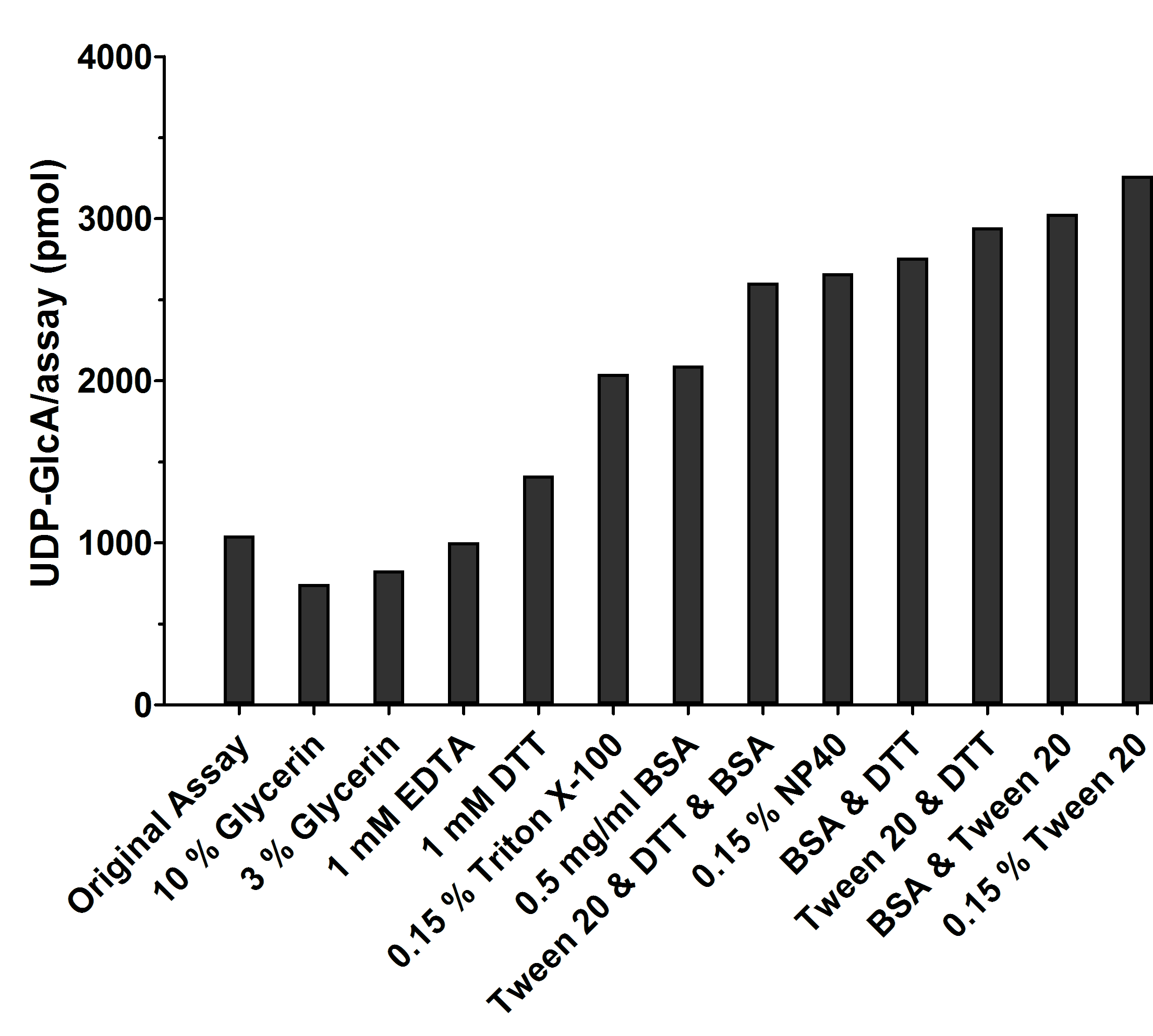

Supplement: Figure S1 — Stabilizing Factors. The addition of stabilizing factors (0.5 mg/ml BSA, 3% Glycerin, 10% Glycerin, 1 mM DTT, 1 mM EDTA, 0.15% Triton X-100, 0,15% Tween20, 0,15% NP40, 1 mM Zn2+) increase DrGK activity in a coupled HPLC enzyme assay with 4 hours incubation time. (TIF) [file pone.0089690.s001.tif]

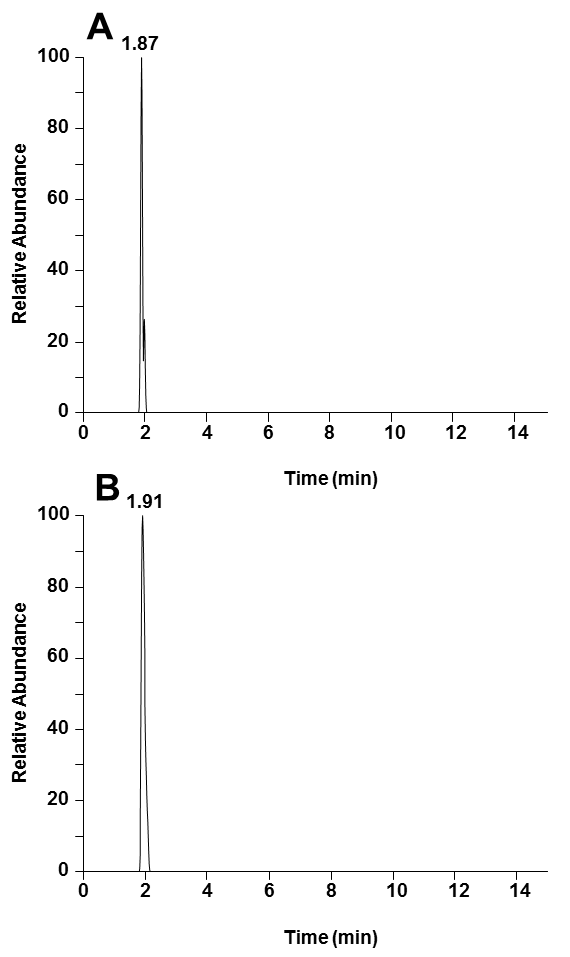

Supplement: Figure S2 — LC/MS Measurement. We also performed a LC/MS measurement of the UDP-GlcA produced during the 70 hours standard HPLC enzyme assays and confirmed that the mass of the product signal (m/z = 579.03) is identical to commercial UDP-GlcA reference compound. (A) 70 hours standard HPLC enzyme assay measurement (B) 50 µM UDP-GlcA as reference compound. (TIF) [file pone.0089690.s002.tif]
