# Supplementary material for: Molecular Cloning of a Novel Glucuronokinase/Putative Pyrophosphorylase from Zebrafish Acting in an UDP-Glucuronic Acid Salvage Pathway
Source: PLoS One. 2014 Feb 28;9(2):e89690. doi: 10.1371/journal.pone.0089690 (PMC3938481; doi:10.1371/journal.pone.0089690)
Supplement: Table S1 — Primer sequences used to make expression constructs for the different protein domains. (DOCX) [file pone.0089690.s003.docx]

| Gene of Interest  (Expression System) | Size  [bp] | Primer | Sequence | T_A_  [°C] | Restriction Site |
| --- | --- | --- | --- | --- | --- |
| DrGKUP | 1874 | DrGKUP_Xba_F | 5’-agtctagaATGATTTGCATACTTTTAGTTGCAG-3’ | 57 | Xba I |
| (*N. benthamiana*) |  | DrGKUP_Sal_R | 5’-atgtcgacTGTTCTGACCCTGTACTGATGG-3’ |  | Sal I |
| DrUP | 803 | DrGKUP_Xba_F | 5’-agtctagaATGATTTGCATACTTTTAGTTGCAG-3’ | 57 | Xba I |
| (*N. benthamiana*) |  | DrUP_Sal_R | 5’-atgtcgacCAAGCCATTTAGTGTAATCAG-3’ |  | Sal I |
| DrGK | 1094 | DrGK_Xba_F | 5’-agtctagaATGCTTGCTCACTACTCTTCAAAGC-3’ | 57 | Xba I |
| (*N. benthamiana*) |  | DrGKUP_Sal_R | 5’-atgtcgacTGTTCTGACCCTGTACTGATGG-3’ |  | Sal I |
| DrGKUP | 1869 | DrGKUP_Nco_F | 5’-atccatggTTTGCATACTTTTAGTTGC-3’ | 55 | Nco I |
| (*E. coli*) |  | DrGKUP_Sal_R | 5’-atgtcgacGTTCTGACCCTGTACTGATGG-3’ |  | Sal I |
| DrGKUP | 1874 | DrGlcAK_Sac_F | 5’-atgagctcGTTTGCATACTTTTAGTTGCAGG-3’ | 52 | Sac I |
| (*S. cerevisiae*) |  | DrGlcAK_Sal_R | 5’-atgtcgacATCAGTTCTGACCCTGTACTGATG-3’ |  | Sal I |
| XtGKUP | 1874 | XtGKUP_Xba_F | 5’-agtctagaATGATTTGTATCTTGCTAGTGGC-3’ | 57 | Xba I |
| (*N. benthamiana*) |  | XtGKUP_Sal_R | 5’-tagtcgacTCTGTCCACCTGTGTTTCC-3’ |  | Sal I |
